# Complications of Long COVID: Unraveling a Case of Very-Late-Onset Myasthenia Gravis

**DOI:** 10.7759/cureus.70552

**Published:** 2024-09-30

**Authors:** Hamid Feiz, Courteney Castellano, Leyla Feiz

**Affiliations:** 1 Internal Medicine, Nova Southeastern University Dr. Kiran C. Patel College of Allopathic Medicine, Fort Lauderdale, USA; 2 Osteopathic Medicine, Nova Southeastern University Dr. Kiran C. Patel College of Osteopathic Medicine, Fort Lauderdale, USA

**Keywords:** covid-19, late-onset myasthenia gravis, post-covid complications, post-infectious myasthenia gravis, sars-cov-2 (severe acute respiratory syndrome coronavirus -2)

## Abstract

Myasthenia gravis (MG) is defined as an autoimmune neuromuscular disorder where autoantibodies disrupt synaptic transmission at the neuromuscular junction by targeting the acetylcholine receptor (AChR), muscle-specific kinase (MuSK), or other proteins related to the AChR complex. This disruption leads to characteristic muscle weakness and rapid fatigability. Clinically, MG is classified based on the age of onset into three distinct categories: early-onset MG (younger than 50 years), late-onset MG (between 50 and 64 years), and very-late-onset MG (65 years and older). We present a rare case of an 81-year-old man who presented with dysarthria, shortness of breath, diplopia, and oropharyngeal dysphagia to both solids and liquids for approximately seven days and was noted to be more progressive in the last 48 hours prior to his presentation to the emergency room. Upon arrival at the emergency room, he complained of shortness of breath and diplopia. Of note, approximately four months prior to this admission, he was diagnosed with COVID-19 pneumonia and was treated appropriately with remdesivir and corticosteroids. He had an uneventful COVID-19 pneumonia hospitalization and was discharged home. Given the progressive nature of his symptomatology, particularly dyspnea, he was transferred to the ICU for further evaluation and treatment. Laboratory results were positive for AChR binding, blocking, and modulating antibodies, confirming the diagnosis of MG. The patient received treatment consisting of pyridostigmine, a pulse dose of methylprednisolone, and intravenous immunoglobulin (IVIG) therapy. This case is unique and highlights a case of a very late onset of MG and the manifestation of new-onset MG four months following COVID-19. Additionally, this patient had a very delayed onset of MG symptoms, as he presented four months after his infection with COVID-19, compared to the average onset of reported cases of post-COVID MG being four to eight weeks post-infection with COVID-19. This uniquely delayed onset, occurring beyond a three-month window post-COVID-19 infection, aligns with the criteria established by the Centers for Disease Control and Prevention (CDC) and the World Health Organization (WHO) for a diagnosis of “Post-COVID Condition,” also known as “Long COVID.” This case illustrates the intricate link between post-viral states and autoimmune responses, particularly in geriatric patients. The pathophysiology linking COVID-19 to MG primarily involves immune dysregulation triggered by the viral infection, which may disrupt immune tolerance and lead to clinical autoimmunity. This case stresses the need for vigilance in diagnosis and managing neurological complications in the context of viral respiratory illnesses, particularly in vulnerable populations.

## Introduction

Myasthenia gravis (MG) is uncommon; the overall prevalence ranges from 150 to 250 cases per million individuals and an estimated annual incidence of eight to 10 cases per million persons per year [1]. This autoimmune disorder is characterized by the presence of autoantibodies against the nicotinic acetylcholine receptor (NAChR) [2]. The underlying mechanism involves the disruption of the neuromuscular pathway, resulting in diminished stimulation of the muscles, manifesting as muscle weakness that worsens with exertion, known as fatigable weakness. Symptoms can be generalized or focal, with common involvement of the ocular and bulbar regions, and in severe cases, may lead to respiratory compromise [3].

Variants of MG are classified based on various factors, including autoimmune mechanisms, targeted molecules within the skeletal muscle, thymic status, genetic attributes, response to treatment, and the clinical presentation of the disease. Among patients who have MG with acetylcholine receptor (AChR) antibodies, the age at onset follows a bimodal distribution pattern, supporting the use of a cutoff age of 50 years to distinguish between early-onset and late-onset disease [4]. Recently, those with the onset of myasthenic symptoms after 65 years of age have been categorized as having very-late-onset MG [5].

It is well-established that viral infections can trigger new onset of neurological disorders with autoimmune etiologies [6]. COVID-19 is a respiratory illness caused by SARS-CoV-2 [2]. New-onset MG has been described as a rare neuromuscular complication of SARS-CoV-2 infection [6]. However, recognizing and managing new-onset MG following a viral infection can pose challenges, particularly concerning respiratory symptoms stemming from hypoventilation due to muscle weakness or hypoxia [7]. Notably, the literature review revealed only seven reported cases of very-late-onset MG post-COVID-19 in patients ages 65 or older. Of these seven cases reported, only our patient developed new-onset MG symptoms three months following a COVID-19 infection, thus meeting the criteria established by the Centers for Disease Control and Prevention (CDC) and the World Health Organization (WHO) for a diagnosis of “Post-COVID Condition,” also known as “Long COVID” [8]. In this paper, we present a case report of an elderly male with no previous autoimmune disease history who developed a very-late-onset MG with a longer than average timeline of MG symptom onset following an infection with COVID-19.

## Case presentation

An 81-year-old man was evaluated for new-onset dysarthria and presented with symptoms of labored breathing. Alongside exacerbated previous complaints, he reported new-onset diplopia and dysphagia to both liquids and solids. Past medical history includes hypertension, obstructive sleep apnea, hypercholesterolemia, ischemic cardiomyopathy with three stent placements, and prostatic cancer under remission. Notably, he had been hospitalized previously for pneumonia due to COVID-19 four months prior to this admission, where he was treated with remdesivir and corticosteroids. On physical examination, the patient exhibited right palpebral ptosis, which notably improved following a two-minute application of an ice pack. He was noted to also have dysarthria and dysphonia.

**Figure 1:**
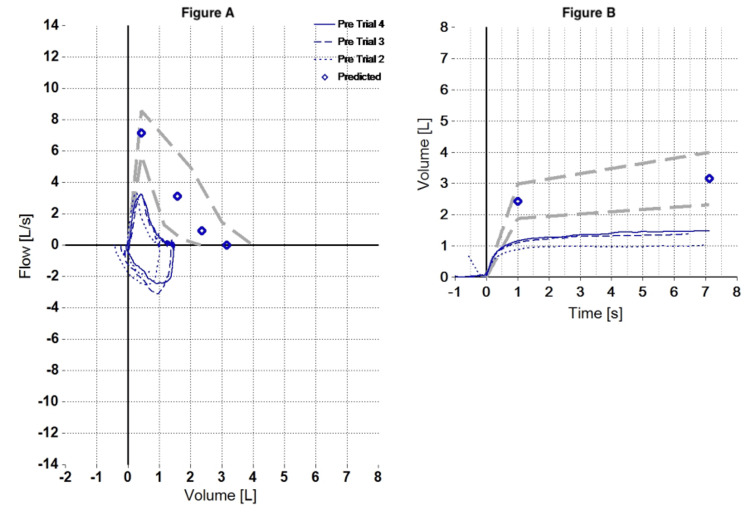
Bedside spirometry with a restrictive pattern (z-score), forced vital capacity (FVC): 15.8 cc/kg. Report according to American Thoracic Society/European Respiratory Society (ATS/ERS) parameters (2022) [9]. The information provided indicates the results of a spirometry test with a restrictive pattern and the specific value of forced vital capacity (FVC) as 15.8 cc/kg. Patient weight is 71.4kg. (A) Spirometry flow/volume curve. (B) Spirometry volume/time curve.

The patient was admitted with acute respiratory failure. A complete blood count and arterial blood gas on admission showed normocytic anemia and mild hypoxemia (Table 1 and Table 2). Given high suspicion for MG, AChR antibodies were sent for further analysis (Table 3). A bedside spirometry reported a restrictive pattern with FVC 15.8 cc/kg, as shown in Table 4 and Figure 1.

**Table 1:** Initial complete blood count Abnormal values are presented in bold.

Lab	Value	Reference range
Hemoglobin (g/dL)	11.4	14-18
Hematocrit (%)	33.2	37-50
Mean corpuscular volume (fL)	92	80-99
White blood cell count (cells/uL)	5.60 × 10^3^	4.00-10.00 × 10^3^

**Table 2:** Initial arterial blood gas analysis Abnormal values are presented in bold.

Lab	Value	Reference range
pH	7.37	7.35-7.45
Partial pressure of oxygen (mmHg)	55	83-108

**Table 3:** Laboratory test for rule-out myasthenia gravis Abnormal values are presented in bold.

Lab	Value	Reference range
Acetylcholine receptor binding antibody (nmol/L)	11.60	Negative: < or = 0.30 nmol/L; equivocal: 0.31-0.49 nmol/L; positive: > or = 0.50 nmol/L
Acetylcholine receptor blocker antibody (%)	27	<15% inhibition
Acetylcholine receptor modulating antibody (%)	82	<32% inhibition

**Table 4:** Bedside spirometry *Indicates value outside normal range or significant post change. FEF25-75: forced expiratory flow between 25% and 75% of the FVC; FEV1: forced expiratory volume in one second; FET: forced expiratory time; FIVC: forced inspiratory vital capacity; FVC: forced vital capacity; LLN: lower limit of normal; PEF: peak expiratory flow; PIF: peak inspiratory flow; Pred: predicted value; %Pred: predicted value percentage

Parameter	Pred	LLN	Pre-best	Trial 4	Trial 3	Trial 2	%Pred	z-Score
FVC (L)	3.15	2.31	1.49*	1.49*	1.39*	1.02*	47	-3.26
FEV1 (L)	2.42	1.87	1.16*	1.16*	1.10*	0.88*	48	-3.76
FEV1/FVC	0.781	0.679	0.777*	0.777*	0.791*	0.859	100	-0.06
FEF25-75 (L/s)	2.38	0.96	0.99*	0.99*	0.98*	1.06*	42	-1.60
PEF (L/s)	7.15	-	3.33	3.24	3.25	3.33	47	-
FET (s)	-	-	7.1	7.1	6.5	6.9	-	-
FIVC (L)	3.15	2.31	1.61*	1.55*	1.61*	1.45*	51	-3.02
PIF (L/s)	-	-	3.13	2.46	3.13	2.55	-	-

Given the significant worsening of his dyspnea and vital capacity (15 cc/kg), the patient was electively intubated for ventilatory support. After initiating ventilatory support, the patient received treatment consisting of pyridostigmine, a pulse dose of methylprednisolone, and intravenous immunoglobulin (IVIG) therapy.

**Figure 2:**
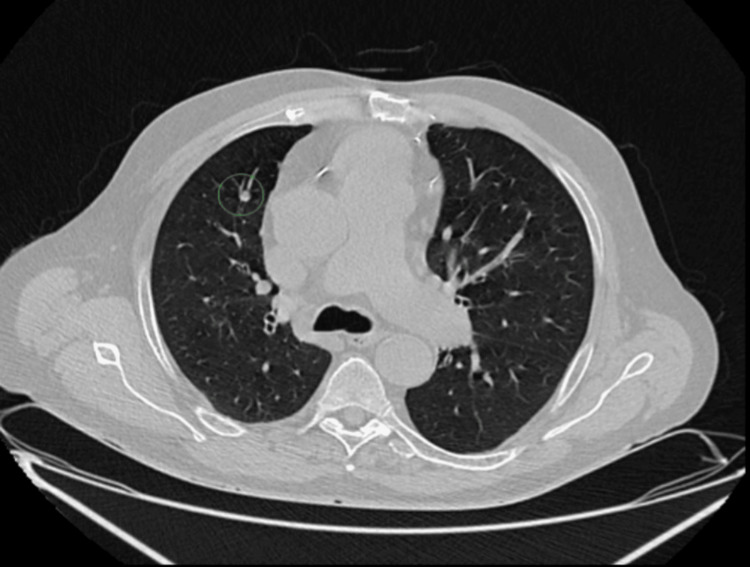
Axial chest CT scan without contrast shows solid nodule with regular and well-defined edges in the upper lobe of the right lung (indicated by green circle). Compared with previous CT scans dated September and January 2022, evidencing stability of the findings of previous results

In addition to the observed positive changes, imaging studies showed no evidence of thymoma or thymus hyperplasia, as shown in Figure 2. Furthermore, there were no significant differences between the CT conducted upon admission and the patient's previous chest imaging results.

Other laboratories showed positive results for AChR binding, blocking, and modulating antibodies, confirming the diagnosis of MG (Table 2). The patient was subsequently extubated and discharged on prednisone 20 mg four times a day, mycophenolic acid 500 mg twice a day, pyridostigmine 60 mg three times a day, and home nursing care. The patient was closely followed up on by neurology post-discharge. His mycophenolic acid was titrated to 1,000 mg every 12 hours with marked improvement in his ventilatory mechanics. At the current 12-month follow-up appointment, the patient's MG has remained asymptomatic without any recurrence of his MG symptoms.

## Discussion

In most populations, the age at onset of AChR MG has a bimodal pattern, characterized by a lower peak at 30 years of age and a higher peak at 70-80 years of age [1]. Therefore, our patient’s symptoms at 81 years old can be classified as very late MG. To the best of our knowledge, there has only been one other reported case of bulbar MG triggered by COVID-19 infection. Because this presentation is unusual, we conducted a comprehensive review of such reported cases with very late MG and summarized our findings in Table 5.

**Table 5:** Summary of previously published cases of very-late-onset myasthenia gravis presenting after SARS-CoV-2 infection *Indicates a case of very-late-onset myasthenia gravis (age of patient > 65 years old). AChR, acetylcholine receptor antibody; AF, atrial fibrillation; AZT, azathioprine; F, female; IVIG, intravenous immunoglobulin; M, male; MG, myasthenia gravis; MR, mitral regurgitation; MuSK, muscle-specific kinase; NICM, non-ischemic cardiomyopathy; OSA, obstructive sleep apnea; PE, pulmonary embolism

Study	Age/gender	Comorbidities and family history	COVID-19 symptoms	CT chest	Duration between COVID-19 and MG (in days)	MG presentation	Thymus pathology	Antibody	MG treatment	Outcome
*Assini et al., 2020 [10]	77/M	None	Dyspnea and fever	Bilateral interstitial pneumonia	56	Chewing difficulty, dysphonia, diplopia, and eyelid ptosis, worsened by muscular activity	None	Anti-MuSK	Failure to pyridostigmine; AZT	Improved
Bhandarwar et al., 2021 [11]	61/M	Bronchial asthma and diabetes mellitus	Not reported	CT severity score of 13/25 with no evidence of any mediastinal mass	56	Generalized MG/dysphagia, dyspnea, generalized weakness	Present (new from prior CT done at the time of the COVID-19 infection	Anti-AchR	IVIG, corticosteroids, pyridostigmine, thymectomy	Complete recovery
*Chatterjee et al., 2022 [4]	83/M	AF, NICM, TN, moderate MR	Acute respiratory failure due to pneumonia	Mild emphysematous changes	30	Generalized muscle weakness, respiratory failure	None	Anti-AChR	Failure to pyridostigmine; AZT + steroids	Dead (from other medical causes)
Essajee et al., 2021 [12]	7/F		Multisystem inflammatory syndrome in children (MIS-C)	-	28	Generalized MG/proximal muscle weakness, waddling gait, and compensatory lumbar lordosis; fatigable bilateral ptosis	No	Anti-AChR	IV methylprednisolone and IVIG, 2 g/kg, followed by oral prednisone that was gradually weaned over a four-week period	Improved
Huber et al., 2020 [13]	21/F	Family history of Hashimoto's thyroiditis, Addison's disease, and pernicious anemia	Mild respiratory symptoms, aching limbs and head without fever, accompanied by anosmia/ageusia; dry eyes and nasal mucosa	Not reported	14	Ocular MG	None	Anti-AChR	IVG and pyridostigmine	
*Jõgi et al., 2022 [14]	65/M	Hypertension, hypercholesterolemia, cataract	Pneumonia	Not reported	14	Generalized muscle weakness, diplopia, and dysphagia	None	Anti-AChR	First treatment: prednisolone + IVIG; second treatment: AZT	Improved
Karimi et al., 2021 [15]	38/F	Not reported	Myalgia, fatigue, coughing, and fever	Mild bilateral lower lobe opacities in the lung without thymoma	28	Generalized MG/diplopia, ptosis, fatigue, and dysphagia	No	Anti-AChR	Pyridostigmine and prednisolone	Improved
Karimi et al., 2021 [15]	57/M	Chronic heart failure (CHF) and implantable cardioverter-defibrillators (ICD) since 10 years ago	Dry cough, fatigue, and fever as high as 38.5°C	Indicated signs of COVID-19 pneumonia in the lower lobe of the lung	7	Generalized MG/diplopia, ptosis, fatigue, and dysphagia	No	Anti-AChR	Pyridostigmine and prednisolone	Improved
Karimi et al., 2021 [15]	61/F	Not reported	Mild signs of respiratory problems	Demonstrated thymoma	42	Generalized MG/dysphagia, nasal speech, ocular ptosis, diplopia, proximal muscle weakness, dyspnea	CT chest showed thymoma	Anti-AChR	Plasma exchange, pyridostigmine bromide, and prednisone	Improved
Muhammed et al., 2021 [16]	24/F	Not reported	Flu-like symptoms	Revealed no thymoma	28	Generalized MG/bilateral fatigable ptosis, complex ophthalmoplegia, symmetrical lower motor neuron facial weakness, dysarthria. She had weakness and fatigability in all four limbs	No	Anti-MuSk	Pyridostigmine with unsatisfactory clinical response, followed by azathioprine	Not reported
*Muralidhar et al., 2021 [17]	65/M	Diabetes, hypertension	Fever, cold, and cough	Normal	42	Generalized muscle weakness	None	Anti-AChR	IVIG; prednisolone, AZT, and pyridostigmine	Improved
Pérez et al., 2020 [18]	48/M	Paranoid schizophrenia, under treatment with aripiprazole; inverse psoriasis	High fever, dry cough, dyspnea, and myalgia 15 days previously	Right lower lobe opacification and lingula consolidation	10	Ocular/binocular diplopia	None	Anti-AChR	No specific MG-directed therapy given	Improved
Restivo et al., 2020 [19]	68/M	Not reported	Fever	Normal	7	Generalized muscle weakness, diplopia, and dysphagia	None	Anti-AChR	Pyridostigmine and prednisone	Improved
*Restivo et al., 2020 [19]	71/F	Not reported	Fever and cough	Bilateral interstitial pneumonia	5	Bilateral ocular ptosis, diplopia, and dysphagia	None	Anti-AChR	Plasmapheresis; hydroxychloroquine	Improved
*Sriwastava et al., 2020 [5]	65/F	Left RCC, pituitary adenoma, pulmonary carcinoid, meningioma, history of prior PE	Diarrhea, myalgia, and fatigue	Not reported	11	Left eye ptosis, diplopia with vertical up-gaze after 20 seconds; mild eye closure weakness	None	Anti-AChR	Pyridostigmine	Improved
Taheri et al., 2022 [20]	35/F	Not reported	Dyspnea, myalgia, and the sore throat	Peripheral consolidation and ground glass opacity	21	Generalized MG/severe weakness in upper/lower limbs, blurred vision, and droopy eyelids	None	Anti-AChR	Pyridostigmine	Improved
*Our case	81/M	Hypertension, OSA, hypercholesterolemia, ischemic cardiomyopathy, and prostatic cancer under remission	Pneumonia	Sub-segmentary atelectasis in both lower lung lobes	120	Right palpebral ptosis, respiratory insufficiency	None	Anti-AChR	Methylprednisolone + pyridostigmine; IVIG	Improved

In our brief literature review, we reviewed 17 other cases of new, very-late-onset MG triggered by COVID-19 infection. There is no definitive expected latent period between the onset of COVID-19 infection and the development of MG symptoms. Our review of the reported cases revealed a gap between acute COVID-19 infection and the onset of MG symptoms ranging between five days and eight weeks. In contrast, the present case study describes a patient who developed MG four months subsequent to the resolution of COVID-19 infection and the associated symptoms, thereby indicating a potential delayed onset of MG post-COVID-19.

Of the symptoms documented in cases of new-onset MG following infection with COVID-19, ocular involvement (ptosis and diplopia) was the most common, being found in six out of 17 of the cases in our literature review. There has been only one other reported case by Jõgi et al. of new-onset bulbar symptoms, such as slurred speech, dysphagia, and dysphonia, following an infection with COVID-19 [14]. The case reported by Jõgi et al. was a younger patient of 65 years of age who also developed difficulty with speech and slurring of words five days following pneumonia from COVID-19 infection [14]. Our case is unique in that the onset of MG symptoms occurred very late in life, at 81 years of age, and the symptoms of dysarthria and oropharyngeal dysphagia did not present until four months following his COVID-19 infection.

Both genetics and environmental factors play crucial roles in the development of MG. Certain genes, such as HLADRB1*1501, HLADQ5, and CTLA4, have been specifically linked to MG and its subgroups [1]. Unfortunately, we do not have confirmation of these genes in our patients. However, as we mentioned earlier, in elderly patients without a prior history of autoimmune diseases, environmental factors exert a strong influence on the development of MG. Additionally, associations between MG and other viral infections, including the West Nile virus, Zika virus, and the novel coronavirus (COVID-19), have been reported [4,21-23]. Gilhus et al. mentioned that less than 50% of the cases are due to genetic causes, suggesting infections as the major external cause of MG [23]. Until the last decade, the environmental factors contributing to MG were largely unknown. Various hypotheses have posited the involvement of the Epstein-Barr virus in the thymic pathology observed in patients diagnosed with MG; however, subsequent studies with control groups have not corroborated these findings [24-25]. 

MG patients are more vulnerable to contracting COVID-19 and are prone to experiencing adverse outcomes compared to individuals without this autoimmune condition [2]. This susceptibility can be attributed to the weakened immune systems of MG patients resulting from immunosuppressive therapy, immune system dysregulation, as well as respiratory muscle weakness possibly leading to respiratory failure [2]. Infection with COVID-19 is also likely to cause an acute exacerbation in MG patients due to the activation of the immune system leading to the secretion of pro-inflammatory cytokines and molecules inducing cytokine storm, acute respiratory distress syndrome (ARDS), and multi-organ failure [2]. 

The underlying mechanism behind a new-onset MG after a COVID-19 infection is not completely understood and needs further investigation. This mechanism can be linked to various factors involved in the inflammatory response of the virus. There is a similar structure between the AChR and SARS-CoV-2 receptor. Because of this molecular mimicry between SARS-CoV-2 and the AChR, COVID-19 has the potential to initiate an immune response that produces antibodies targeting the ACh receptor [2]. COVID-19 infection also significantly increases the likelihood of provoking acute exacerbations in individuals with MG. Specifically, COVID-19 in MG patients increases the risk of myasthenic crisis, respiratory failure, and permanent pulmonary damage [3]. 

Our patient was treated with high-dose pyridostigmine 60 mg three times a day and a pulse dose of methylprednisolone. Although most patients can be successfully treated with this intervention [26], it is known that IVIG is the first-line treatment for patients with deterioration associated with certain acute and chronic neuroimmune diseases [27]. Due to the increase in the severity of his dyspnea and detrition of mechanical ventilatory function with vital capacity of 15 cc/kg, the decision of elective intubation was made to provide ventilatory support while the patient received appropriate treatment. In the ICU, he received appropriate ventilatory support and IVIG therapy, which led to the resolution of his symptoms and the improvement of his ventilatory function. He was extubated and discharged home three days post-extubation. He remains asymptomatic and in remission 12 months after initial diagnosis on mycophenolic acid 1,000 mg twice a day and pyridostigmine 60 mg three times a day.

## Conclusions

The currently available literature has provided evidence of new-onset presentation and exacerbation of MG due to viral infections. Furthermore, the continuously updated information about the complications and consequences of COVID-19 infection has narrowed the diagnostic gap in this very-late-onset presentation of MG. This case highlights the potential for the development of new-onset MG in a patient over 80 years old, who belongs to a very small group in which MG appears to be a chronic consequence of infections caused by respiratory viruses such as COVID-19 and influenza. Given the anticipated increase in COVID-19 cases, it is essential to emphasize the ongoing significance of post-infection follow-up consultations and evaluations of respiratory function tests as an integral component of the management plan.
